# Rabih El Chammay: addressing mental health in crises

**DOI:** 10.2471/BLT.23.031223

**Published:** 2023-12-01

## Abstract

Rabih El Chammay talks to Gary Humphreys about his journey in mental health, the importance of mental health in humanitarian emergencies, and the opportunity within crises to kick-start mental health system reform.

*Q: You studied medicine at the Saint Joseph University of Beirut. At what point did you decide to specialize in psychiatry?*

A: When I started to work with patients in a psychiatric hospital in Lebanon in 2002. I was utterly fascinated, especially by the conversations I had with individuals living with severe mental disorders. They offered insights into both the complexity and fragility of the human mind – topics that continue to absorb me. I studied in Paris for two years at the Sainte Anne Hospital, where I was exposed to cognitive behavioural therapy and psychoanalysis, and the more I studied, the more questions I had, particularly in regard to the way the mind reflects and relates to its environment. This is an interest that has only deepened with my study of public mental health and, more recently, Buddhist philosophy.

*Q: When did you develop your interest in public mental health?*

A: In 2006, I volunteered with some non-governmental organizations who were helping in the support of people displaced as a result of the war between Lebanon and Israel. That experience brought me into close contact with many people who were clearly very distressed, and brought home to me the limits of a pure clinical, disease-based treatment model. There were obviously mental health needs, but it wasn’t clear to me how traditional clinical care could meet them. So I started asking myself questions, and read the Inter-Agency Standing Committee guidelines for mental health and psychosocial support (MHPSS) in complex emergencies with great interest when they were published in 2010, noting their emphasis on the interplay between mental health and psychosocial factors. Then I went on to pursue a Master in mental health policy and service development with NOVA University in 2014. I came out of that master’s with a strong sense of the extent to which mental health relates to and is embedded in context – interwoven with social determinants such as gender, poverty, stigma, violence, displacement, access to education, access to healthcare, etc. And it became clear to me that what many patients needed, including the patients I had encountered in my early years, was not the latest medication – although access to effective medication is of course important – or institutional care; what they needed was to be part of a community, to be supported to carry on with their lives according to their wishes, and to have access to the care they needed near their homes in the community. This applies to people in normal life as well as to people in humanitarian crises, and I say that having been through the experience of conflict-related displacement myself.

“It is important not to medicalize or psychologize human suffering.”

*Q: When was this?*

A: This was in 1984 and I was six years old. I still remember it vividly. Bombs started falling on our village in the south of Lebanon. We had to clear out of our home in minutes. I still recall how fast my parents needed to make decisions, and how much energy and focus they needed in the first few weeks to make sure that my siblings and I had a safe place to sleep, and enough food and water. In such times, you need to have the clarity of mind to make the decisions you need to make, and the energy to follow through. Had they been extremely distressed by the events, or experiencing depression, anxiety or any other disorder without access to appropriate support and care, or basics like shelter, food and water, they would not have been able to care for themselves and for us the way they did. My conviction that the meeting of basic needs is essential to meeting mental health needs in crises is partly informed by that experience. Which is not to say that access to basic necessities is always enough, because psychosocial support may also be required, for example in the form of psychological first aid.

*Q: What do you mean by psychological first aid?*

A: Psychological first aid is used to provide psychological support in the immediate aftermath of crisis events. It is typically described as offering humane, supportive and practical assistance, with the aim of helping people manage their distress and helping them access the things they need. It can be provided by any front-line health worker, without having to rely on mental health professionals, who will not always be present. It’s important to note that debriefing – getting people to talk through their experience, something that used to be at the heart of what was known as critical incident stress management – is no longer used and is indeed considered to be potentially harmful.

*Q: What has replaced debriefing?*

A: A readiness to listen. People need to know that we are there to listen if they want to speak, but that it is totally fine if they prefer not to. No one should be pushed to tell their story if they are not ready to do so. The underlying aim of psychological first aid is to empower the person by encouraging existing coping skills, and giving them the tools they need to cope. Of course, for some people, this is not enough and referrals to specialist services may be needed. However, as I say, such specialist help may not be available, and it is important to realize that much can be achieved by non-specialists with the right training.

*Q: How have you applied these approaches to addressing the needs of people in Lebanon?*

A: It’s an interesting question, and it’s perhaps worth taking a moment to reflect on the crises the population has had to deal with. They include civil war, the influx of more than 1.5 million displaced Syrian people in 2011, an ongoing political and economic crisis which started in 2019, the COVID-19 (coronavirus disease 2019) crisis, and in August 2020 the Beirut port explosion, one of the most powerful non-nuclear explosions of modern history. All of this has had a profound impact on the mental health of the population. And now we are seeing the effects of the ongoing conflict in Gaza and the risk of a spillover into Lebanon. Through all of this we have been steadily reforming our mental health system, emphasising MHPSS and community-based care. Indeed, as counterintuitive as it might seem, I would argue that crises have been the main drivers of mental health system reform in the country.

*Q: In what way?*

A: It started with the creation of the national mental health programme in the ministry of public health. The seeds of that programme were planted with the MHPSS situation analysis that I did for the humanitarian response to the Syrian crisis in Lebanon that was commissioned by UNHCR (United Nations Office of the High Commissioner for Refugees) in 2013. One of the main findings of that report was the lack of any coordination mechanism or lead agency for the provision of MHPSS. I was also working with WHO (World Health Organization) and having discussions with Dr Walid Ammar, the former director-general of the health ministry, about potentially supporting the ministry in scaling up its mental health role.

“Crises have been the main drivers of mental health system reform in [Lebanon].”

All of this led to the setting up of the national mental health programme in 2014 with the support of WHO, UNICEF (United Nations Children’s Fund) and the International Medical Corps. The programme was tasked with leading the coordination of the newly established MHPSS group and with developing a strategy for mental health in Lebanon. In 2015, the first national strategy for mental health 2015–2020 was launched and implemented, and it has now been renewed for 2030. Without the first MHPSS situation analysis, prompted by the Syrian refugee crisis, I’m not sure it would have happened. Of course, Lebanon is not the first nor the only country to have used a crisis to reform its mental health system, as reported in *Building back better: sustainable mental health care after emergencies*, which was published by WHO in 2013. The bottom line is that humanitarian responses and the funding that comes with them can create momentum and opportunities to reform mental health systems, and good mental health systems can make better use of the scarce resources allocated for mental health in humanitarian emergencies.

*Q: What have been the main achievements of the national mental health programme so far?*

A: It has made us much quicker to respond to crises. For example, we were very effective in developing and implementing a response to the Beirut port explosion and now, as we speak, we are fully engaged in implementing an emergency preparedness plan for any potential full-scale military attack on Lebanon. It’s also worth mentioning that Lebanon was the first country to develop and implement an intersectoral MHPSS response to COVID-19. None of this is intended to argue that the response to all crises should be MHPSS plans. On the contrary, MHPSS plans should be deeply contextualized and rooted in the specificity of each emergency. Of course, people with mental health conditions should have access to proper care, and it goes without saying that humanitarian crises, and economic crises, increase the number of people who might need services, and those people should have timely access to quality services. However, I would argue that it is important not to medicalize or psychologize human suffering, especially in humanitarian emergencies, and especially those caused by armed conflicts. After the Beirut port explosion and still today, when asked about what they need most for their well-being, most people who were affected answer, “Truth and justice”. To me this suggests that addressing mental health needs cannot be reduced to a series of technical or medical challenges. It is bigger than that, encompassing things like security, connectedness, dignity, justice, freedom from oppression, discrimination, etc, and *also* access to good health and mental health care services. Addressing the root causes of people’s mental suffering, as challenging as this might be, can be more validating and more healing for people than psychological support in the traditional sense, especially to those groups who are most oppressed and most disempowered.

